# Thermal Liquid Biopsy (TLB) Focused on Benign and Premalignant Pancreatic Cyst Diagnosis

**DOI:** 10.3390/jpm11010025

**Published:** 2020-12-31

**Authors:** Sonia Hermoso-Durán, Guillermo García-Rayado, Laura Ceballos-Laita, Carlos Sostres, Sonia Vega, Judith Millastre, Oscar Sánchez-Gracia, Jorge L. Ojeda, Ángel Lanas, Adrián Velázquez-Campoy, Olga Abian

**Affiliations:** 1 Instituto de Investigación Sanitaria Aragón (IIS Aragón), 50009 Zaragoza, Spain; shermosod@gmail.com (S.H.-D.); guillermogarcia7@hotmail.com (G.G.-R.); ceballos.laita@gmail.com (L.C.-L.); carlossostres@gmail.com (C.S.); millastrej@gmail.com (J.M.); alanas@unizar.es (Á.L.); 2 Joint Units IQFR-CSIC-BIFI, and GBsC-CSIC-BIFI, Institute of Biocomputation and Physics of Complex Systems (BIFI), Universidad de Zaragoza, 50018 Zaragoza, Spain; svega@bifi.es; 3 Servicio de Digestivo, Hospital Clínico Universitario Lozano Blesa (HCULB), 50009 Zaragoza, Spain; 4 Centro de Investigación Biomédica en Red en el Área Temática de Enfermedades Hepáticas y Digestivas (CIBERehd), 28029 Madrid, Spain; 5 SOTER BioAnalytics, Enrique Val, 50011 Zaragoza, Spain; oscar.sanchez.gracia@gmail.com; 6 Department of Statistical Methods, Universidad de Zaragoza, 50009 Zaragoza, Spain; jojeda@unizar.es; 7 Department of Medicine, University of Zaragoza, 50009 Zaragoza, Spain; 8 Fundación ARAID, Gobierno de Aragón, 50009 Zaragoza, Spain; 9 Departamento de Bioquímica y Biología Molecular y Celular, Universidad de Zaragoza, 50009 Zaragoza, Spain; 10 Instituto Aragonés de Ciencias de la Salud (IACS), 50009 Zaragoza, Spain

**Keywords:** pancreatic cysts, thermal liquid biopsy, differential scanning calorimetry, diagnosis, generalized linear models

## Abstract

Background: Current efforts in the identification of new biomarkers are directed towards an accurate differentiation between benign and premalignant cysts. Thermal Liquid Biopsy (TLB) has been previously applied to inflammatory and tumor diseases and could offer an interesting point of view in this type of pathology. Methods: In this work, twenty patients (12 males and 8 females, average ages 62) diagnosed with a pancreatic cyst benign (10) and premalignant (10) cyst lesions were recruited, and biological samples were obtained during the endoscopic ultrasonography procedure. Results: Proteomic content of cyst liquid samples was studied and several common proteins in the different groups were identified. TLB cyst liquid profiles reflected protein content. Also, TLB serum score was able to discriminate between healthy and cysts patients (71% sensitivity and 98% specificity) and between benign and premalignant cysts (75% sensitivity and 67% specificity). Conclusions: TLB analysis of plasmatic serum sample, a quick, simple and non-invasive technique that can be easily implemented, reports valuable information on the observed pancreatic lesion. These preliminary results set the basis for a larger study to refine TLB serum score and move closer to the clinical application of TLB providing useful information to the gastroenterologist during patient diagnosis.

## 1. Introduction

During recent years, the detection of pancreatic cysts has become more frequent due to improvements in abdominal imaging techniques. The incidence of this pathology is approximately 2% in the adult population [1]. Computed tomography (CT) scans are reported detection between 1.2% and 2.6%, and magnetic resonance imaging (MRI) has even a higher detection capability, ranging between 13.5% and 19.9% [1,2]. The management of these incidentally detected pancreatic cysts is still a challenge, because, even though the risk of being malignant is low, the prognosis in case of pancreatic adenocarcinoma and intraductal papillary mucinous neoplasms- (IPMN)-related pancreatic adenocarcinoma is very poor and has not improved recently [3]. Thus, distinguishing between benign and malignant cysts is difficult, and very often requires surgical intervention with considerable morbidity and mortality. Since 2005, some guidelines have been published and updated: the American Society for Gastrointestinal Endoscopy [4], international consensus guidelines by the International Association of Pancreatology (Sendai guidelines) [5], the American College of Gastroenterology [6], and the International Association of Pancreatology (Fukuoka guidelines) [7,8]. More recently, The European Study Group on Cystic Tumors of the Pancreas published an update, replacing the 2013 European Consensus Statement Guidelines [9].

From a clinical point of view, the classification according to the prognosis of the lesion is: (1) cysts with malignant potential (mucinous), (2) cysts without malignant potential, and (3) malignancies. However, most studies provide a description of biomarkers for differentiating the mucinous and non-mucinous type of cysts. The non-mucinous group comprises serous cystadenomas (SCAs) (the most common type), pancreatic pseudocysts (PCs), and a variety of rare cysts (benign epithelial, lymphoepithelial, congenital, and squamoid cysts). Most are found incidentally and none of them represents a risk for becoming malignant [10]. On the contrary, the mucinous group, including mucinous cystic neoplasms (MCNs) and IPMNs, constitutes the majority of neoplastic premalignant cysts identified in the pancreas, and a precise diagnosis technique would be vital for the management of patients [11]. The development of techniques with greater pre-surgical diagnostic precision would make it possible to avoid the morbidity and mortality associated with a high-risk intervention when it is not strictly necessary, as well as reduce the healthcare overload derived from unnecessary outpatient follow-up in cystic lesions without the potential for malignancy.

Cystic fluid markers become especially relevant when transabdominal ultrasonography, CT or MRI are inconclusive. In these cases, it is necessary to employ another risk predictor to indicate surgery as the most appropriate treatment given the estimated risk.

The presence of amylase at high concentration in cyst fluid indicates that there is a communication between the cyst and the ductal system. This occurs in both pseudocysts and IPMN lesions. When amylase levels are lower than 250 U/L, communication with the conduct can be discarded, with a specificity of 98% [12]. However, amylase value alone is not enough to differentiate between mucinous/non-mucinous or MCN/IPMN, which is important from the point of view of patient management, in deciding whether surgical resection or cyst time-monitoring is recommended [13]. A previous episode of pancreatitis can be of help in distinguishing a pseudocyst from an IPMN lesion (occasionally related to pancreatitis) [14].

Carcinoembryonic antigen (CEA) is also employed as a biomarker. This is a set of highly related glycoproteins involved in cell adhesion, mucin being one of them. It can be used to distinguish between cysts with (MCNs and IPMNs) and without (SCAs and PCs) mucinous epithelium [10]. The major inconvenience of CEA is the absence of an appropriate cutoff value [12]. The current accepted value to classify a cyst as mucinous is CEA > 192 ng/mL [15].

In order to improve diagnosis, studies related to the identification of new biomarkers in cystic fluid or serum have been recently reported [16,17,18,19,20,21].

In 2007 Chaires et al. [22] described the application of differential scanning calorimetry (DSC) in diagnosis using plasma/serum samples from cancer patients. Since then, many studies have confirmed the potential clinical use of this technique, not only applied to plasma/serum [23,24,25,26,27,28,29,30], but also to other biological human samples such as cerebrospinal fluid [31,32]. In 2018 our group coined the name “thermal liquid biopsy” (TLB) for DSC applied to cancer diagnosis and cancer patient’s treatment monitoring [33,34]. The TLB thermogram reports the global denaturation profile for all the proteins present in the serum/plasma sample and the influence of potential interactions between blood plasma proteins and metabolites, therefore reflecting any alteration induced by a certain disease. As in the case of plasma or serum, cystic fluid is also composed of a mixture of proteins and TLB may also be applied as a clinical diagnosis tool. In this work, the potential of TLB as a clinical biomarker for cyst classification has been pursued. In this pilot study, 20 cyst fluid samples were analyzed and their TLB cyst profiles were obtained, with the purpose of finding a correlation between the TLB thermogram and the type of cyst. The proteomic analysis also allowed a description of the more abundant proteins in the cyst fluid, as well as the post-translational modifications present in those proteins. TLB was also applied to serum samples in some of the patients, and TLB serum profile differences between groups was studied to try to determine whether or not differences observed in cyst fluid TLB correlated with serum TLB. This would be extremely important because, in case cyst features are reflected in certain serum alterations, a simple and risk-free plasma/serum TLB analysis could be employed for cyst diagnosis/classification. Despite the low number of samples considered in this pilot study, different patterns in cyst fluid and plasma from patients with pathology could be observed.

## 2. Materials and Methods

### 2.1. Subjects and Samples

Cyst liquid and serum samples from patients with cystic lesions in the pancreas detected by transabdominal ultrasonography or CT or MRI were referred to the Department of Digestive Endoscopy at the Hospital Clínico Universitario Lozano Blesa (HCULB), Zaragoza, Spain, between January 2016 and September 2018. The procedure was in accordance with the recommendations of the local ethics committee and all patients gave their informed consent. Pancreatic cystic fluids were collected by EUS-guided fine needle aspiration (FNA). The EUS-FNA procedure was performed with an Olympus^®^ 140 curvilinear echo-endoscope. Boston Scientific^TM^ Expect^®^ 19 or 22-gauge needles were used depending on the cystic endosonographic features. We noted the characteristics of the aspirated cystic fluid: volume, color, and viscosity. The majority of the fluid was examined by the same cytopathologist for every patient and the rest (at least 1 mL Eppendorf for each patient) was collected for detection of biochemical markers, DSC measurements, and proteomic studies described in this manuscript. The collected cystic fluid samples were then stored at −80 °C until they were prepared for analysis.

Serum samples from healthy subjects as control group (HC) consisted of 85 serum samples from Spanish Caucasian subjects, apparently cancer-free, from the FISABIO (Fundación para el Fomento de la Investigacion Sanitaria y Biomedica de la Comunitat Valenciana) biobank with a homogeneous distribution, including gender (53% men and 47% women), with an average age of 45.2 ± 14.2.

### 2.2. Thermal Liquid Biopsy (TLB) Profile Determination

DSC thermograms were measured using a high-sensitivity differential scanning VP-DSC microcalorimeter (MicroCal, Malvern-Panalytical, Malvern, UK). Cystic liquid samples, serum samples, and reference solutions were properly degassed and carefully loaded into the cells to avoid bubble formation. The baseline of the instrument was routinely recorded before the experiments. Experiments were performed in cystic liquid samples (diluted 1:10 in phosphate buffered saline, PBS) and serum samples (diluted 1:25 in PBS) at a scanning rate of 1 °C/min. Thermograms were baseline-corrected and analyzed using software developed in our laboratory implemented in Origin 7 (OriginLab, Northampton, MA, USA).

### 2.3. Data Analysis

We have developed a phenomenological model in which the TLB serum thermogram is deconvoluted into several individual transitions, modeling each individual transition by the logistic peak or Hubbert function [30,33]. This model has been successfully applied in the analysis of serum samples from melanoma and gastric and lung cancer patients [30,34,35]. From this multiparametric analysis, a TLB serum score (between 0 and 1) can be calculated reporting the level of alterations in plasma (TLB serum score < 0.5, absence of alterations; TLB serum score > 0.5, presence of alterations).

The Kolmogorov-Smirnov test was performed to assess the normal distribution of the variables. Medians between two independent groups were compared with the Wilcoxon test, in non-normal distributions. Averages between two independent groups were compared with the *t*-test, in normal distributions.

### 2.4. Protein Sample Preparation and Protein Identification and Quantification by Mass Spectrometry

Protein concentration: Measured by Bradford protein assay (Bio-Rad, Madrid, Spain) using purified bovine serum albumin (BSA) (10 mg/mL, New England BioLabs, EVRY cedex, France) in PBS as standard. Absorbance at 595 nm of two dilutions from each serum sample was measured in triplicate in a Synergy HT multimode microplate reader (BioTek Instruments, Winooski, VT, USA).

In solution digestion: Samples were evaporated and resuspended in 10 μL of denaturing buffer (6 M urea, 100 mM Tris buffer pH 7.8). Next, cysteines were reduced with 1.5 μL DTT (200 mM) for 30 min at 37 °C and alkylated with 6 μL of iodoacetamide (200 mM) for 30 min in the dark. Unreacted iodoacetamide was consumed adding 6 μL of the reducing agent (200 mM DTT) for 30 min at room temperature. Samples were diluted with 50 mM ammonium bicarbonate to a urea final concentration lower than 1 M. Trypsin digestion (Gold Trypsin, Promega, Madison, WI, USA) was carried out overnight at 37 °C at a 1:20 enzyme/protein ratio. Reaction was stopped adding concentrated formic acid (Merck KGaA, Darmstadt, Germany). Samples were evaporated, resuspended in 2% acetonitrile (ACN), 0.1% formic acid, and filtered through 0.45 μm filters.

Protein identification by LC-ESI-MS/MS: Protein identification was performed on a nano-LC 2D system (LC 425, Eksigent Ekspert TM, Dublin, CA, USA) coupled to a hybrid triple quadrupole/linear ion trap mass spectrometer (4000 QTRAP, Sciex, Foster City, CA, USA). On-line pre-concentration and desalting of samples was performed using a C18 trap cartridge (Luna^®^ 0.3 mm id, 20 mm, 5 µm particle size, Phenomenex, CA, USA) at 10 µL/min for 5 min. Peptide separation was performed using a C18 column (Gemini^®^ 0.3 mm id, 150 mm, 3 μm particle size, Phenomenex, CA, USA), at 5 µL/min of flow rate. Column was maintained at 35 °C. The elution gradient was from 5 to 35% ACN (0.1% formic acid) in 90 min. The mass spectrometer was interfaced with an ESI source (Turbo V™) using a 25 µm ID hybrid electrode and was operated in the positive ion mode. MS source parameters were as follows: capillary voltage 5000 V, de-clustering potential (DP) 85 V and curtain and ion source gas (Nitrogen) 15 psi. Analyses were performed using an information dependent acquisition (IDA) method with the following steps: single enhanced mass spectra (EMS, 400–1400 *m*/*z*) from which the 5 most intense peaks were subjected to an enhanced product ion [EPI (MS/MS)] scan. Protein identification was carried out using the Mascot search engine (Matrix Science; London, UK) and the non-redundant SwissProt database (553,655 sequences; 198,177,566 residues). Search parameters were monoisotopic mass accuracy, peptide mass tolerance ±0.5 Da, fragment mass tolerance ±0.3 Da; one allowed missed cleavage; allowed fixed modification carbamido-methylation (Cys), and variable modification oxidation (Met). Positive identification was assigned with Mascot scores above the threshold level (*p* < 0.05), with at least two identified peptides with a score above homology

Protein SDS electrophoresis: Samples mixed with NuPAGE LDS Sample buffer (Invitrogen), and heated at 95 °C for 4 min, were analysed by sodium dodecyl sulphate–polyacrylamide gel electrophoresis (SDS–PAGE) using 10% acrylamide resolving gels and 4% acrylamide stacking gels (Bio-Rad). The gels were fixed with a mixture of ethanol, acetic acid, and deionized water (40:10:50) for 1 h. After washing in water for 5 min, the gels were stained with Coomassie Brilliant Blue R250 (0.1% in 25% methanol, 10% acetic acid) and de-stained by incubation in 30% acetic acid and 20% methanol. Molecular weights were estimated by comparison with the migration rates of standard proteins (Bio-Rad).

## 3. Results

### 3.1. Clinical Sample Description

Patients who underwent endoscopic ultrasonography procedure were included in this work. A total of 20 subjects, 60 and 40% men and women, respectively, with an average age of 62 ± 13 years.

Based on imaging and cytopathology, the pancreatic cysts were classified into different categories (Table 1).

Clinical information of the samples is detailed in Table 2. All the cysts were between 2 and 15 cm in size and they were located in any region in the pancreas. According to clinical data (amylase and CEA concentrations), samples were divided in two groups: benign cysts (PC, WOPN and SC) and premalignant cysts (IPMN and MCN). There are two samples that turned out not to be cysts, but malignant lesions (PDAC).

Pancreatic pseudocysts (PC) are pockets of fluid, common sequelae of acute pancreatitis or chronic pancreatitis. PCs are important in terms of management and differentiation from other cystic processes or masses in this region. According to the updated Atlanta classification [36], there are two main groups of mature-well defined fluid collections associated with acute pancreatitis: A/Fluid collections in interstitial edematous pancreatitis (PC), and B/Fluid collections in necrotizing pancreatitis (WOPN). Both PC and WOPN were considered benign cysts. From our PC samples, PC 1 and 4 were in the context of acute pancreatitis, and PC 5 was in the context of chronic pancreatitis. The WOPN cysts had the biggest size, between 3 and 15 cm. Both types of pancreatic collections (PC and WOPN) were amylase positive (above 250 U/L) and CEA negative (below 192 ng/mL).

Serous cysts (SC) are benign neoplasms composed of numerous small cysts that are arrayed in a honeycomb-like formation and most individual cysts are typically <10 mm.

Lymphocele (LYM), also known as cystic lymphangioma, is a rare disease. There are no typical clinical manifestations, and most patients were diagnosed incidentally during imaging or surgery. Therefore, diagnosis is challenging. Surgical resection is still considered as the most effective approach for lymphocele, and prognosis is favorable. In our study, SC and Lym were 5 cm in size, and amylase and CEA negative.

Intraductal papillary mucinous neoplasms (IPMN) are epithelial pancreatic cystic tumors of mucin-producing cells that arise from the pancreatic ducts. They are most commonly seen in elderly patients, with sex distribution roughly balanced, a possible slight male predominance. IPMNs are slow growing tumors that have malignant potential and distinct variants have been described: main duct (IPMN 6 and 7), branch duct (IPMN 1, 3 and 4), and mixed branch and main duct (IPMN 2 and 5). Main duct IPMNs have a very high rate of malignancy (up to 70% in reported surgical series [8]); for this reason, the usual recommendation is surgical removal of the affected portion of the pancreas. Branch duct IPMNs are cystic neoplasms of the pancreas that have malignant potential and their management is challenging; the risk of surgery must be carefully weighed against the risk of malignancy when deciding on surgical removal or surveillance. This is the reason why great efforts are taken to distinguish mucinous cysts from other cyst lesions (specially, main duct IPMNs). All IPMNs are considered as premalignant cysts. They had the smallest size, between 2 and 3.5 cm. Four were amylase positive (above 250 U/L) and all were CEA positive (above 192 ng/mL or very closed in case of IPMN3 with 156 ng/mL).

Mucinous Cystadenoma (MCN) is another type of mucinous cystic neoplasm of the pancreas, traditionally considered typical of middle age females. MCN1 was 3 cm in size, amylase and CEA positive.

### 3.2. Analysis of TLB from Cystic Liquid Samples

**Figure 1:**
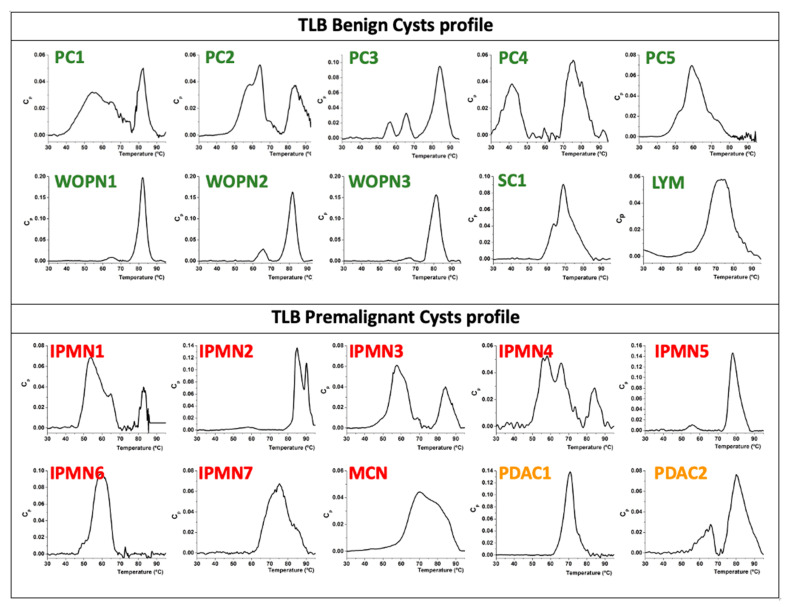
Individual TLB thermograms from cystic liquid samples. Samples were clustered according to their benign or premalignant nature.

TLB thermograms of 20 cystic fluid samples were obtained. Protein concentrations and dilutions could be considered, but in this case TLB curves were normalized according to their area under the curve values (AUC); therefore, signals from the different samples can be compared and uncertainties in protein concentration (inherent to colorimetric methods) are avoided. TLB cyst profiles clustered according to their clinical assessment (benign or premalignant nature) are represented in Figure 1.

**Figure 2:**
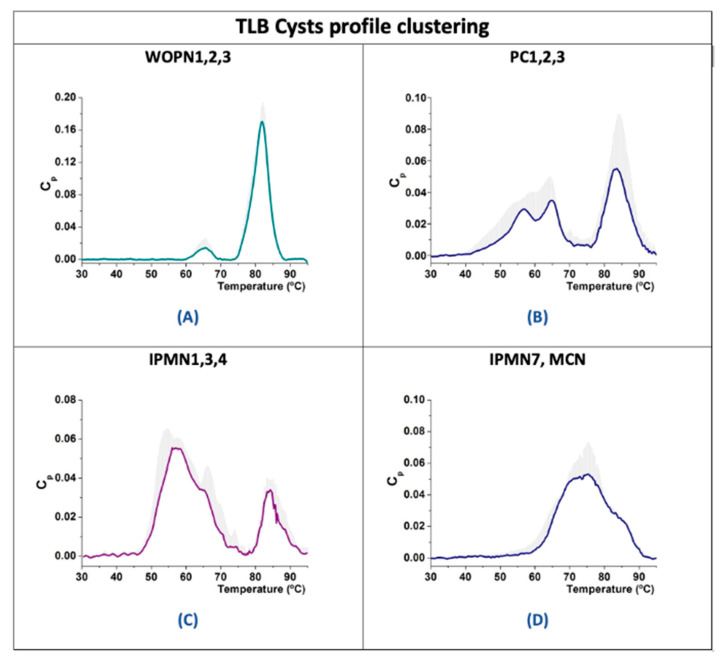
TLB thermograms from cystic liquid samples clustered according to their type. Average curves (colored lines) and standard deviations of curve values (grey) are represented: WOPNs (**A**), PCs (**B**), IPMNs (**C**) and IPMN7/MCNs (**D**).

Regarding the benign cystic group, the WOPN group exhibited a very similar cyst thermogram profile with two peaks at 65 and 82 °C. We can easily distinguish this group from the other benign cysts (Figure 2A). PC5 is the only PC lacking the 85 °C peak, and it is the only PC in a chronic pancreatitis context.

In the premalignant cyst group, branch duct IPMNs (IPMN1, IPMN3 and IPMN4) exhibited a similar profile (Figure 2C). Main duct IPMNs (IPMN6 and IPMN7) exhibited a single peak.

### 3.3. Analysis of Proteomic Signatures from Cystic Liquid Samples

**Figure 3:**
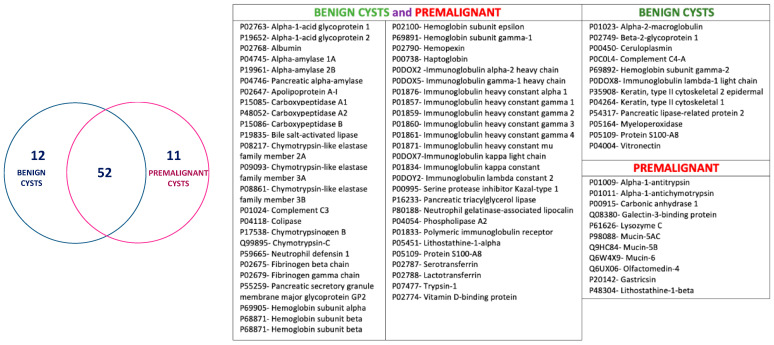
LC-ESI-MS/MS proteomic content of cystic samples. Common proteins were analyzed via Venn diagrams online tool (http://bioinformatics.psb.ugent.be/beg/tools/venn-diagrams). 12 and 11 proteins were detected only in benign cysts (blue) and premalignant cysts (pink), respectively, and 52 proteins appeared in both groups (intersection set). Table comprises the detailed information of the proteins in each set.

Proteins identified by LC-ESI-MS/MS (detailed in Table S1) were analyzed and clustered according to the benign or premalignant nature of the cyst (Figure 3). This first classification distinguishes 52 proteins common in the cyst groups, and 12 and 11 proteins present only in benign cysts and premalignant cysts, respectively. A deeper analysis according to different groups of cysts was performed.

#### 3.3.1. Benign Cysts

**Figure 4:**
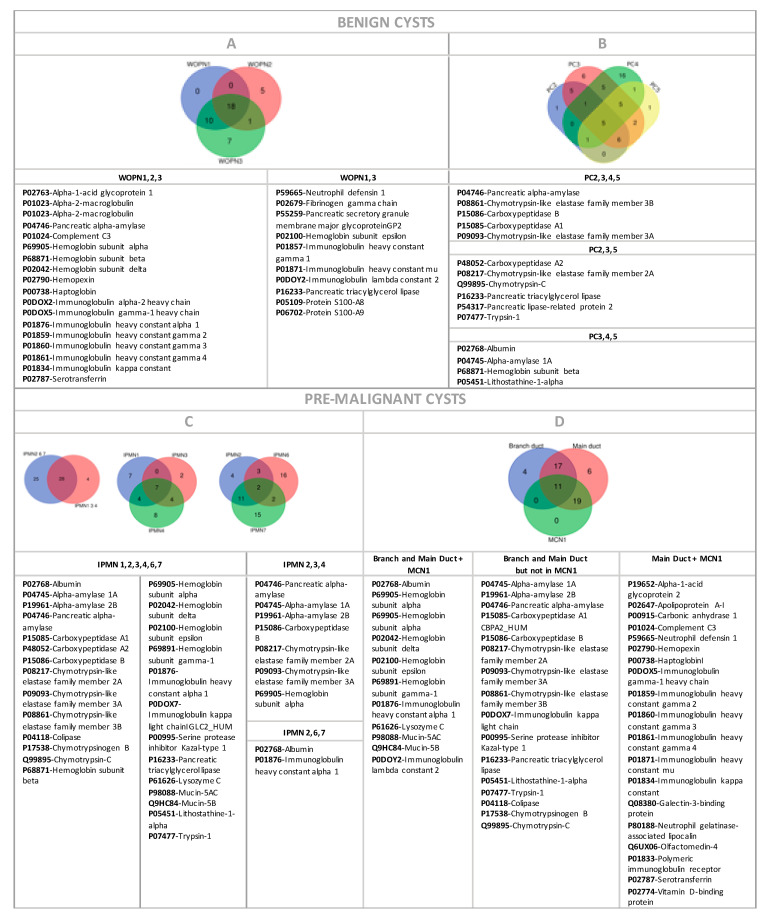
LC-ESI-MS/MS proteomic content of cystic samples according to cystic types: (**A**) WOPN, (**B**) PC, (**C**) IPMN, and (**D**) MCN+IPMN. Common proteins were analyzed via Venn diagrams online tool. Colors code for different groups and numbers inside each set and shared sub-sets indicate the number of identified proteins.

The WOPN cyst group exhibited a homogeneous proteomic profile (Table S1). 18 out of 41 (44%) proteins were shared by all cysts in this group (Figure 4A). The similarity in these samples was even higher, because 10 more proteins were common in WOPN1 and WOPN3. Low protein concentration in WOPN2 (Figure S1A) could prevent proper identification of more proteins in that sample. The most abundant proteins found in this group were globulins (macroglobulin and immunoglobulins), a type of protein related to immunological response as a consequence of an inflammation process. This is consistent with the nature of this specific type of cyst: walled-off pancreatic necrosis (WOPN) is a well-circumscribed area of necrosis which occurs as a late complication of acute pancreatitis, generally after four weeks from the initial episode. Singular proteins detected in WOPN1 and WOPN3 samples were S-100 proteins. They belong to the S100 protein family, having important roles in inflammation and may also be useful markers for gut inflammation [37]. Once secreted in the extracellular space, S100A9 acts as a chemo-attractant, recruiting further inflammatory cells and creating an inflammatory microenvironment that promotes tumor development [38].

Common proteins of this group detected by electrophoresis (Figure S1A) are: proteolytic proteins, albumin, glycoside hydrolases, metalloproteases, immunoglobulins, and elastases. This agreed with the proteomic profile previously detailed (Table S1).

The homogeneity found in the proteomic signature of this group was reflected in the TLB cyst profiles of the cystic liquid (Figure 2A). As could be observed, compared to the rest of cystic groups studied in this work, these TLB cyst profiles represent a particular signature for WOPN cysts, easily distinguishing this group from the rest. They showed two peaks at T_1max_ ≈ 65 °C and T_2max_ ≈ 81 °C, with a peak width at around 10 °C. Denatured proteins included in these peaks are detailed in Figure 4A.

In the PC cyst group, proteomic profiles data was also collected (Table S1), except for PC1, for which no protein was detected, except albumin. There are 5 proteins shared by all PCs (Figure 4B) and 10 proteins are shared by at least three PCs: PC2, PC3 and PC5 had 6 common proteins and PC3, PC4 and PC5 4 proteins. These cysts were negative for globulins (macroglobulin and immunoglobulins), indicating there were not any inflammatory processes going on (except for PC4, the rest of PCs in this subgroup were not in an acute pancreatitis context). The PC4 cyst, in acute pancreatitis context, exhibited 17 proteins not found in any other PCs (Table S1). It also contained a small number of carboxypeptidase or pancreatic elastase related proteins, absence of pancreatic triacylglycerol lipase and trypsin-1 and presence of globulins (macroglobulin and immunoglobulins), serotransferrin and lacto-transferrin, protein S100-A9, neutrophil defensin 1, and myeloperoxidase.

Common proteins of this group were detected in electrophoresis (Figure S1B) (except for PC1, where the total amount of protein was very low): glycoside hydrolases and metalloproteases (all PCs), proteolytic proteins (PC3 and PC4), albumin (all but PC2), lipases (all but PC5), immunoglobulins (PC3 and PC4) and elastases (PC2 and PC3). This was consistent with the proteomic profiles.

TLBs of PC cystic liquid (Figure 2B) were similar for PC1, PC2 and PC3, with three peaks at T_1max_ ≈ 55 °C, T_2max_ ≈ 65 °C (two close peaks) and T_3max_ ≈ 85 °C (peak width around 10 °C). Common proteins were found in these samples: amylase, IgGA, carboxypeptidase pancreatic elastase, chymo-trypsinogen, trypsin, glycoprotein GP2 and phospholipase A2. In PC5, T_3max_ ≈ 85 °C was missing, and this could be the result of the absence of any of these proteins: IgGA, glycoprotein GP2, phospholipase A2 and chymo-trypsinogen. In PC4 there was a shift in the transitions, from 55 to 40 °C and from 85 to 75 °C (peak width was maintained in both cases).

In this group the clinical explanation for these differences could lie in the pancreatitis context for samples PC4 (acute) or PC5 (chronic).

#### 3.3.2. Premalignant Cysts

The IPMN cyst group was more homogeneous from the clinical point of view. IPMN was the cystic group in this study with a number of proteins identified in the proteomic profile (Table S1). For example, it was not possible to identify proteins for IPMN5. One interesting observation was that none of the IPMNs contained globulins (macroglobulin or immunoglobulins), or they were negligible in other cyst groups, and only IPMN7 clearly contained them. These proteins are related to immunological responses as a consequence of inflammation and, therefore, it seemed that inflammation was not associated with this type of cyst.

Branch duct IPMNs (IPMN1, IPMN3 and IPMN4) shared 28 proteins with main duct IPMNs (IPMN2, IPMN6 and IPMN7) (Figure 4C). Branch duct IPMNs shared seven common proteins, and main duct IPMNs shared only two proteins. Neither of these proteins were unique in any of the two groups (they were included in the 28 proteins in common).

Common proteins of this group detected in the electrophoresis gel are albumin and immunoglobulins (IPMN1, IPMN2 and IPMN5); glycoside hydrolases and elastases (IPMN1, IPMN3, IPMN4 and IPMN5); metalloproteases (IPMN1 and IPMN3); and lipases (IPMN3 and IPMN4). Proteolytic proteins were not present in any of IPMNs samples (Figure S1D). This was consistent with the proteomic profiles.

TLBs of IPMN cystic liquid (Figure 2C) were similar for IPMN1, IPMN3 and IPMN4, all being branch duct IPMNs. They exhibited two close peaks (around T_1max_ ≈ 55 °C, T_2max_ ≈ 65 °C) and a third peak at T_3max_ ≈ 85 °C (peak width around 10 °C). These peaks could correspond to the common proteins found in this group (amylases, carboxypeptidase and pancreatic elastases).

TLB of main duct type IPMN cystic liquids had a wider single peak at ≈55 °C and ≈75 °C for IPMN6 or IPMN7, respectively.

TLB of mixed type IPMN cystic liquids presented characteristics from both branch or duct IPMN groups with a low signal peak at ≈55 °C and one peak at ≈80 °C for IPMN5, or two peaks at ≈85 °C and 90 °C for IPMN2.

There was no proteomic profile available for IPMN5, but, according to electrophoresis, there was no protein from the amylase family, and additionally IPMN 2 and IPMN 7 lacked amylases, carboxy-peptidase and pancreatic elastases. These three cysts did not exhibit any peak (or it was neglectable) at ≈55 °C.

The MCN 1 cyst was in the mixed cysts group, but it had a premalignant nature.

MCN1 shared 11 proteins with IPMNs, either branch or main duct type, (Figure 4D) and 19 more proteins in common only with main duct IPMNs (Figure 4D) with five of these protein shared with IPMN7. When comparing MCN1 and IPMN7 profiles (Table S1), 15 common proteins were found. In fact, IPMN7 was the only IPMN cyst showing globulins (macroglobulin and immunoglobulins). In addition, protein profile in electrophoresis for MCN1 was similar to IPMNs (Figure S1E). However, 17 proteins were detected in IPMNs, but not in MCN1. There were no proteins shared only with branch duct IPMNs.

When comparing MCN1 and IPMN7 profiles (Table S1), 15 common proteins were found. In fact, IPMN7 was the only IPMN cyst showing globulins (macroglobulin and immunoglobulins).

Proteins detected in electrophoresis for MCN1 were similar to IPMNs (Figure S1E).

TLB for cystic liquid sample exhibited a wide transition (around 30 °C width) with two peaks (around T_1max_ ≈ 70 °C, T_2max_ ≈ 85 °C), very similar to the IPMN7 profile (Figure 2D). MCN1 did not exhibit any peak at around 55 °C and, again, no amylases, carboxypeptidase or pancreatic elastases were detected by proteomics.

Mixed Cyst Group was a completely heterogenous group comprising a serous cyst, a lymphocele, and two samples of pancreatic ductal adenocarcinoma (PDCA) (which cannot be considered as a cyst).

SC and LYM: The main difference between serous cyst and lymphocele (cystic benign samples), compared to the rest of samples, was the presence of apolipoprotein A-I (only MCN1 and IPMN7 seemed to contain it) (Table S1). In addition, they were positive for globulins (macroglobulin and immunoglobulins), as with the samples with an active inflammatory process. Some typical pancreatic cystic proteins were missing: amylase, metalloproteases, lipases, elastases. Some of these proteins were already employed as clinical biomarkers for pancreatic cyst diagnosis [9], such as amylase.

Common proteins of this group detected in electrophoresis are: serotransferrin, albumin, lipoproteins and immunoglobulins (Figure S1C). This agreed with the proteomic profiles.

All TLB cyst profiles in this group showed particular features and looked different from the other cystic profiles. They exhibited one single peak: SC at T_1max_ ≈ 70 °C (peak width around 20 °C) and LYM at T_1max_ ≈ 75 °C (peak width around 25 °C). Common proteins (Table S1) are albumin, lipoproteins, hemoglobins, globulins, and serotransferrin.

PDACs: Proteomic profiles of PDACs (Figure S2) showed 20 shared proteins from hemoglobin, immune-globulins and transferrin groups. PDAC1 also contained other proteins, and PDAC2 contained proteins we previously identified in cysts, such as amylases, metalloproteases, lipases, elastases.

TLB cyst profile in PDAC1 exhibited one peak (T_1max_ ≈ 70 °C) and PDAC2 showed two peaks (T_1max_ ≈ 65 °C and T_2max_ ≈ 80 °C) (Figure 1). This difference could be related to the different proteomic profile between the two samples mentioned above. Electrophoresis also confirmed this different pattern (Figure S1F).

### 3.4. Analysis of TLB from Serum Samples

**Figure 5:**
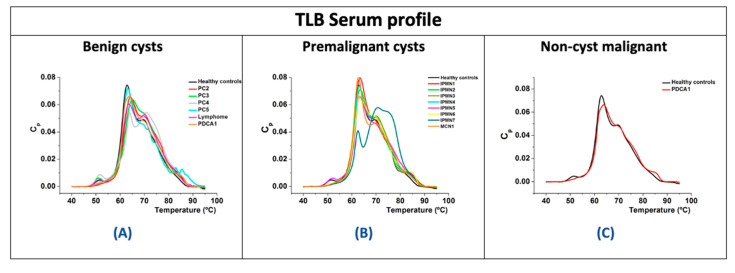
Examples of serum TLB thermograms from different types of cystic patients, clustered according to cyst types: premalignant (**A**), benign (**B**) and non-cyst malignant (**C**). Typical TLB thermogram for a healthy subject is represented with black line in (**A**–**C**).

TLB thermograms from serum samples were obtained. The goal was to search for any potential reflection of the cystic pathology in plasmatic serum. TLB serum profiles were normalized according to their area under the curve values (AUC), again avoiding protein concentration influence. Then, they were clustered according to the clinical assessment of the cyst (Figure 5). We focused our attention on distinguishing between premalignant and benign cysts. Unfortunately, it was not possible to obtain serum samples at the same time as the eco-endoscopy procedure for all the patients included in this study.

PCs are benign lesions found in the context of acute or chronic pancreatitis. It has been previously reported that inflammatory processes can be reflected in TLB serum profile [23]. According to the PC TLB serum thermograms, they seemed to be somewhat different (Figure 5A).

IPMNs, being premalignant lesions, could also exhibit some distinctive features compared to healthy patients in their TLB serum profile on the basis of the previous studies on TLB applied to cancer diagnosis [30,33,34]. Apparently, in the case of IPMNs, TLB serum profiles, except for IPMN7, seem quite similar to healthy controls (Figure 5B).

The multiparametric analysis previously developed in our group [30] was applied to TLB serum profiles. The purpose was to help in the identification of cystic disease-related TLB features and the quantification of the TLB serum score for patients’ serum (Table S2). Mono-variant analysis of the individual TLB parameters obtained from the thermograms showed that 6 out of 15 parameters were statistically different (*p*-value < 0.05). Only these six parameters were used to construct the classification model and calculate the TLB serum score for each sample, as previously described in [33].

**Figure 6:**
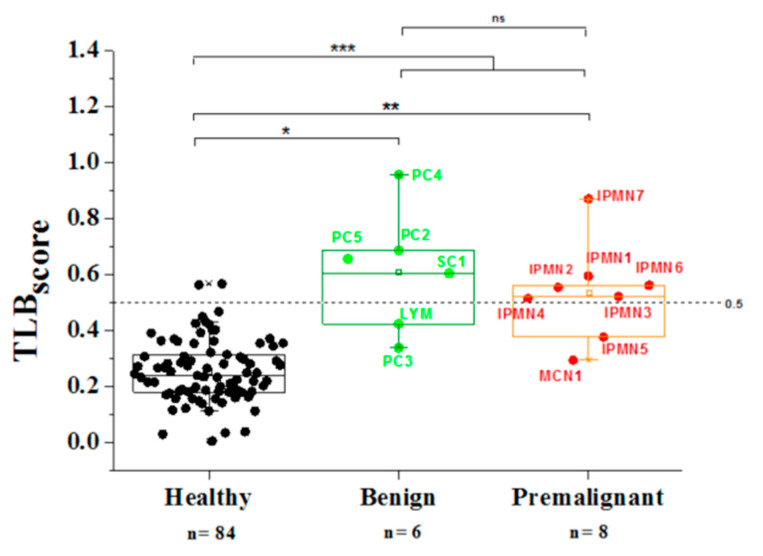
TLB serum score from serum TLB profiles from healthy controls and both types of cysts (benign and premalignant). Median values were compared using the Wilcoxon test: * *p*-value_healthy-benign_ = 0.00026; ** *p*-value_healthy-premalignant_ = 0.000057; *** *p*-value_healthy-cysts_ = 0.0000012; ^ns^ *p*-value_benign-premalignant_ = 0.501.

According to the results in Figure 6, the TLB serum score comparison between healthy subjects and cyst patients (both, benign and premalignant) indicated that the differences were statistically significant using the Wilcoxon test (*p*-value < 0.001). Similarly, TLB serum score could differentiate between healthy subjects and benign cyst patients (*p*-value < 0.001), and between healthy subjects and premalignant cyst patients (*p*-value < 0.001).

TLB serum score values are between 0 and 1: the closer to 0, the smaller the alterations in plasma (healthy status), while the closer to 1, the larger the alterations in plasma (diseased status). TLB serum score values were mainly under 0.5 for healthy (82 out of 84, 98% true negative rate) and over 0.5 for cysts patients (10 out 14, 71% true positive rate). This meant that this score may be useful for detecting the presence of cystic lesion. The area under the ROC curve is 0.94 (Figure S3) with sensitivity of 71%, specificity of 98%, a positive predictive value (PPV) of 83%, and a negative predictive value (NPV) of 98%. Unfortunately, there was no statistical difference between both cysts’ groups; that is, TLB serum score did not discriminate between benign and premalignant cysts (*p*-value = 0.501).

TLB serum values closest to 1 in PC corresponded to those associated to the acute or chronic pancreatitis context (inflammatory process can be reflected in serum).

TLB serum values of IPMN were around 0.5 (except in IPMN7). CEA value for IPMN7 was over 50,000 ng/mL, the highest of all premalignant cysts.

All types of cyst could be clustered differentially from healthy controls by using this single TLB serum score. These results agreed with our previously published results on lung cancer disease [33] in which TLB serum score was able to discriminate between diseased and healthy subjects.

Cystic pathologies are local lesions for which a systemic reflection in blood might not be expected. However, if cystic pathology is accompanied by inflammation, blood alterations may be important, even for a benign lesion.

**Figure 7:**
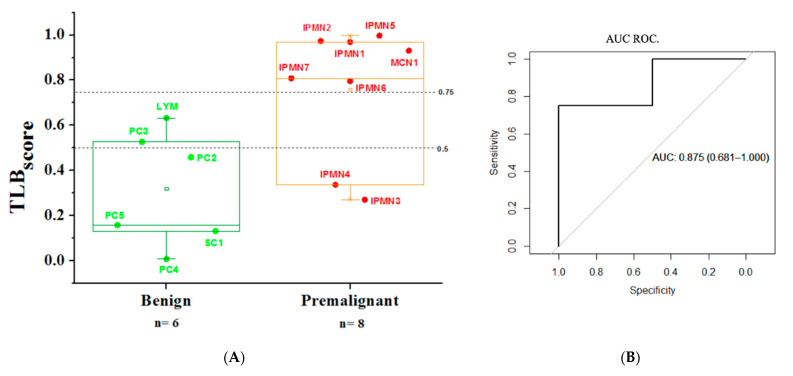
(**A**)/TLB serum score parameters from serum TLB serum profiles from benign and premalignant cyst patients. (**B**)/ROC curve illustrating the statistical performance of TLB serum score (benign vs. premalignant cysts). AUC = Area Under Curve (95%CI).

As a further development, in this study we also proposed to evaluate whether TLB serum score could provide useful information to gastroenterologists for the diagnosis before and besides endoscopic ultrasound procedures. Despite the small number of samples (14 serum samples from cyst patients), a restricted TLB serum score excluding healthy control subjects and considering benign cysts as control samples was performed. Benign and premalignant cysts were 43% and 57% of the samples, respectively. First, we performed a mono-variant analysis of TLB parameters (Table S3) and none of the individual parameters was statistically different (*p*-values > 0.05). Therefore, only parameters presenting *p*-value below 0.25 were considered in constructing the classification model and calculating this new TLB serum score (benign vs. premalignant cysts). For a TLB score threshold of 0.5, 4 out 6 (67%) benign cysts had a TLB score below 0.5, and 6 out 8 (75%) premalignant cysts had a TLB score above 0.5. The area under the ROC curve is 0.875 (Figure 7B) with sensitivity of 75%, specificity of 67%, a positive predictive value (PPV) of 75% and a negative predictive value (NPV) of 67%. When using the Youden index as a threshold (0.75), all benign samples were well classified (TLB score below 0.75).

## 4. Discussion

Pancreatic neoplasms are generally discovered incidentally and consists of IPMNs, predominantly [7]. The diagnosis of malignant IPMN lesions involves a certain degree of subjectivity and variability, which is undesirable for clinical practice, due to the lack of standardized guidelines. Thus, there is a necessity of new diagnosis tools to differentiate between benign and premalignant pancreatic cysts to help in making decisions about surgical intervention or periodic surveillance.

The analysis of cyst fluid may provide information regarding established biomarkers that helps the physician in the diagnosis [13,39]. This type of biological sample includes a diverse amount and type of proteins, representing an interesting challenge for thermal liquid biopsy. TLB for blood serum samples has been proven useful for different types of cancer, and premalignant pancreatic cysts will eventually evolve to pancreatic cancer. Additionally, TLB has been applied to other body fluids (serum, plasma, urine, synovial and cerebrospinal fluids [25,31,40,41], or even tumor digestion), provided that the TLB thermogram is a reflection of the protein composition, interactions, and modifications in the complex sample [32]. Similarities in protein composition of cysts (which is a known biomarker for cyst classification) will result in similarities in TLB cyst profiles, thus, providing the basis for a diagnostic procedure based on TLB. As has been previously described, TLB cyst profile reflects protein composition, but also protein interactions. The presence or absence of a certain protein could promote changes in the profile for other proteins in the sample, especially if they were interacting (interactome concept) [25]. Similar effects could be envisaged for protein modifications as a result of metabolic or pathologic processes.

In our study, we recruited 20 patients who, after being diagnosed with a cystic lesion by CT scan or MNR, underwent routine endoscopic ultrasonography where a sample of cyst fluid was obtained. The final clinical diagnosis of the lesions found was based on the different clinical features of the patient (fluid aspect, biochemical biomarkers and pathological anatomy results) according to current clinical guidelines [8,36].

To our knowledge, this was the first time that pancreatic cyst fluid was characterized using a TLB technique and thermal profiles were clustered according to the clinical information on the cysts. We confirmed that cyst liquid thermograms reflect protein content in the samples. There was a high intra-group variability within the TLB cyst thermograms. Only WOPN cysts could be easily differentiated from PCs, because there was a clear profile type that could be associated with them (Figure 2A) and they also shared 18 proteins (Figure 4A). More samples are required to clearly define a common cyst thermogram for benign or premalignant cysts. Having the TLB thermogram available for specialist appraisal, perhaps the diagnosis could be oriented to the MCN cyst type. As we have already discussed, there is a considerable subjective component in the physician diagnosis, and the more complementary tools available for a better discrimination, the better for the gastroenterologist in producing an appropriate evaluation statement.

Another goal of this study was linking cyst thermograms to proteomic signatures of cyst samples, bearing in mind that, as we said before, TLB cyst profile reflects sample composition and also potential interactions and modifications in proteins. Each protein unfolds in a certain manner and the calorimetric cyst profile obtained is characteristic for that unfolding process. When two or more proteins are together in the same sample, the resulting profile will be the sum of the signal of the independent profiles when proteins are not interacting; or, in case the proteins interact, the resulting profile could change [42].

A deeper analysis of the proteomic profile of pancreatic intra-cyst sample can be found in the literature [43]. We used proteomic identification to assess the correlation between the TLB thermograms and the protein content. The TLB methodology is based on apparently simple curves, but they contain relevant information for diagnosis.

This is clear when looking at WOPN samples (as we already described above): TLB thermograms were quite similar and overlapped, and proteomic profiles also confirmed this similarity. Nevertheless, results are not so clear for the rest of the cyst groups and further studies to increase the number of intra-cyst samples are needed.

This pilot study reveals some interesting aspects. In IPMN, less inflammatory process indicators have been detected, specifically less immunoglobulins in comparison to benign cysts. There were also less proteins related to iron metabolism (serotransferrin, lacto-transferrin, ferritin, hemopexin and haptoglobin) which has been referenced as an indicator of health alteration [44,45].

The information obtained through the cyst TLB thermogram is not focused on the detection of a specific biomarker but comprises information about a large amount of proteins and their relations (interactions and modifications), which could be interesting when doubts about the diagnosis of a cyst are cast. This study represents a proof of concept to confirm that TLB cyst thermograms contain valuable information. According to these preliminary results, a further, larger study can lead to the establishment of certain specific profiles for different type of cysts and can help during diagnosis.

The analysis of a cyst fluid sample implies that the patient has undergone an endoscopic ultrasonography procedure, a risky, invasive test that nowadays is the only way to achieve a reasonably accurate diagnosis of the benign or premalignant nature of cysts. Here we wondered if alterations due to the cyst could have a systemic reflection in blood serum, and whether or not they could be detected through TLB. If metabolic alterations in cyst fluid are translated into blood serum alterations, a quick, low-risk, minimally invasive tool to obtain information on the cyst would be available to differentiate between healthy/cystic patients and/or benign/premalignant cystic patients. TLB has been previously applied in our lab to study different type of tumor disease such as gastric, lung cancer or melanoma [30,33,34]. In the present study, we also obtained 15 serum samples from the 20 patients and their TLB serum profiles were obtained (Figure 5). As normally occurs with TLB serum thermograms, it is difficult to visually discriminate and distinguish among patients’ profiles. In previous studies from our group, we contributed to develop, first, a multiparametric analysis [30] and, later, a TLB serum score [33] to manage and assess TLB thermograms according to a simple interpretation index (TLB serum score from 0 to 1) that could be implemented in diagnosis, easily allowing the stratification of the patients.

We first compared the TLB serum thermograms from patients to thermograms from healthy subjects that were not suffering from the disease. We applied a general TLB serum score previously reported [33] and the results were statistically different when comparing healthy subjects with benign or malignant cyst patients, individually or together as a pooled group of patients (*p*-values lower than 0.05) (Figure 6). Specificity and sensitivity values (98% and 71%, respectively), as well as PPV and NPV values (83% and 98%, respectively), were quite high.

TLB serum scores (from benign or premalignant cyst groups) were not statistically different when using the TLB general formula when comparing to healthy subjects. Therefore, on that basis it was not possible to distinguish between both types of cyst. Therefore, we focused our attention on specifically comparing both patients’ groups and obtaining a cyst-specific TLB serum score that could be applied to evaluate intra-group cyst patient variability. This new TLB serum score would strengthen the discrimination power, based on the specific parameters reflecting differences between cysts. The challenge is considerable, because a mono-variant analysis of cyst TLB parameters (Table S3) showed that none of the individual parameters was statistically different between the benign and premalignant cyst group. As we previously confirmed in our studies, the combination of all the parameters in a multiparametric-based single TLB serum score increased the discrimination ability. Despite the small patient sample cohort, it was possible to distinguish between benign and premalignant cysts. This specific TLB serum score (values from 0 to 1) were over 0.75 (according to Youden) in 6 out of 8 (75%) premalignant samples, while TLB serum scores were below 0.75 in 6 out of 6 (100%) benign samples. The diagnosis accuracy based in the area under the curve (AUC) was 0.875, a promising starting point for extending the study. Therefore, more serum samples from patients with pancreatic cysts should be included in a larger future study, but these preliminary results are promising and allow us to foresee that the TLB serum score could be applied routinely in the clinic as an additional complementary tool helping physicians in making better diagnostic decisions.

## 5. Conclusions

TLB analysis can be applied to both plasmatic serum and cyst fluid as a sort of high information content tool. Despite the small number of samples in this pilot study, it represents a proof of concept for developing a useful technique for classifying and evaluating risk in pancreatic cysts based on liquid biopsy in both body fluids. A future, larger study, with a larger number of samples for each cyst category, could confirm whether TLB of plasma or cyst fluid could be a new diagnosis tool to differentiate between benign and premalignant pancreatic cysts to help clinician gastroenterologists in making decisions about disease management. In case of serum, TLB would represent a quick, low-risk, minimally invasive tool easily translated to clinical practice for diagnosis and patient monitoring. In addition, TLB is reasonably cheap for serum tests (an estimated cost of 100–200 €/$ per test, although with a higher cost for cyst fluid test), and could be performed with predefined frequency for patient surveillance.

## Figures and Tables

**Table 1:** Patient Description.

	Type of Cyst						Non-Cyst Malignant Lesions	
	Benign				Pre-Malignant			
	PC (*n* = 5)	WOPN (*n* = 3)	SC (*n* = 1)	LYM (*n* = 1)	IPMN (*n* = 7)	MCN (*n* = 1)	PDAC (*n* = 2)	Total (*n* = 20)
Age (years) *	63 ± 10	62 ± 10	72 ± 0	52 ± 0	72 ± 13	42 ± 0	40 ± 2	62 ± 13
Male/female %	80/20	67/33	100/0	0/100	71/29	0/100	0/100	60/40

* Average ± standard deviation (sd) PC = pseudocyst; WOPN = Walled-off pancreatic necrosis; IPMN = intraductal papillary mucinous neoplasm; SC= Serous Cyst; MCN= Mucinous Cystadenoma; LYM= lymphocele; PDAC= Pancreatic Ductal Adenocarcinoma.

**Table 2:** Clinical Cyst Sample Description.

Group	Name	Localization in the Pancreas	Cyst Size (cm)	Amylase (U/L)	CEA (ng/mL)	Final Clinical Diagnosis
Benign Cysts	PC1	Body	5.5	>11,000.0	9.23	Pseudocyst (In Acute Pancreatitis Context)
	PC2	Head	4.1	>11,000.0	64.2	Pseudocyst
	PC3	Head	3.5	5635.0	68.4	Pseudocyst
	PC4	Body	15.0	nd	nd	Pseudocyst (In Acute Pancreatitis Context)
	PC5	Head	3.0	>11,000.0	28.8	Pseudocyst (In Chronic Pancreatitis Context)
	WOPN1	Tail	6.4	>11,000.0	2.4	Walled-off pancreatic necrosis
	WOPN2	Head	10.0	>11,000.0	2.0	Walled-off pancreatic necrosis
	WOPN3	Body	4.0	>11,000.0	50.0	Walled-off pancreatic necrosis
	SC1	Body	5.0	41.0	0.7	Serous Cyst
	*LYM*	Head	4.9	24.0	0.8	Lymphocele
Pre-Malignant Cysts	IPMN1	Body	2.6	>11,000.0	489.2	Branch duct IPMN
	IPMN2	Head, Body, Tail	2.0	162.0	1488.0	Main duct IPMN
	IPMN3	Head	2.3	>11,000.0	156.0	Branch duct IPMN
	IPMN4	Head	2.5	>11,000.0	556.0	Branch duct IPMN
	IPMN5	Isthmus	3.5	>11,000.0	225.0	Mixed Branch and Main duct IPMN
	IPMN 6	Head	3.0	10.0	392.0	Main Duct IPMN with pancreatic extension
	IPMN 7	Head, Body, Tail	3.5	4.0	>50,000.0	Main Duct IPMN with pancreatic extension
	MCN1	Body	3.3	3401.0	1617.0	Mucinous Cystadenoma
Non-Cyst Malignant Lesions	PDAC 1	Body, Tail	8.0	nd	nd	Pancreatic Ductal Adenocarcinoma
	PDAC 2	Head	0.5	>11,000.0	1192.0	Pancreatic Ductal Adenocarcinoma

nd = not determined: Amylase < 250 U/L, communication with the conduct can be discarded; CEA > 192 ng/mL to classify a cyst as mucinous.

## Data Availability

The data presented in this study are available on request from the corresponding author.

## Supplementary Materials

The following are available online at https://www.mdpi.com/2075-4426/11/1/25/s1, Figure S1: Electrophoresis analysis of proteomic profiles, Figure S2: LC-ESI-MS/MS proteomic content of cyst samples types identification, Figure S3: ROC curve illustrating the statistical performance of TLB serum score (healthy vs. cysts); Table S1: Detailed information of cyst proteomic profiles, Table S2: Mono-variant analysis of TLB parameters of healthy controls and cysts patients, Table S3: Mono-variant analysis of TLB parameters of benign and premalignant cysts patients.
