# Supplementary material for: Thermal Liquid Biopsy (TLB) Focused on Benign and Premalignant Pancreatic Cyst Diagnosis
Source: J Pers Med. 2020 Dec 31;11(1):25. doi: 10.3390/jpm11010025 (PMC7823923; doi:10.3390/jpm11010025)
Supplement: Supplementary file 1 [file jpm-11-00025-s001.pdf]

Supplementary Materials: Thermal liquid biopsy (TLB) focused on benign and premalignant pancreatic cyst diagnosis

Sonia Hermoso-Durán, Guillermo García-Rayado, Laura Ceballos-Laita, Carlos Sostres, Sonia Vega, Judith Millastre, Oscar Sánchez-Gracia, Jorge L. Ojeda, Ángel Lanas, Adrián Velázquez-Campoy and Olga Abian

Category/Family		Accession Uniprot	Protein description	PC1	PC2	PC3	PC4	PC5	WOPN1	WOPN2	WOPN3	SC	LYM	IPMN1	IPMN2	IPMN3	IPMN4	IPMN6	IPMN7	MCN1	PDCA1	PDAC2
Transport		P02768	Albumin																			
Iron metabolism-related proteins	Hemoglobins	P68871	Hemoglobin subunit beta																			
		P69905	Hemoglobin subunit alpha																			
		P02042	Hemoglobin subunit delta																			
		P69891	Hemoglobin subunit gamma-1																			
		P69892	Hemoglobin subunit gamma-2																			
		P02100	Hemoglobin subunit epsilon																			
	Proteolityc proteins	P00738	Haptoglobin																			
		P02787	Serotransferrin																			
		P02788	Lactotransferrin																			
	Transport-related proteins	P02792	Ferritin light chain																			
		P02790	Hemopexin																			
	Oxidoreductases	P00450	Ceruloplasmin																			
Globulins	Macroglobulins	P01023	Alpha-2-macroglobulin																			
	Immunoglobulins	P01834	Immunoglobulin kappa constant																			
		P0DOX5	Immunoglobulin gamma-1 heavy chain																			
		P01876	Immunoglobulin heavy constant alpha 1																			
		P01857	Immunoglobulin heavy constant gamma 1																			
		P01859	Immunoglobulin heavy constant gamma 2																			
		P01860	Immunoglobulin heavy constant gamma 3																			
		P01861	Immunoglobulin heavy constant gamma 4																			
		P0DOX8	Immunoglobulin lambda-1 light chain																			
		P0DOY2	Immunoglobulin lambda constant 2																			
		P0DOX7	Immunoglobulin kappa light chain																			
		P01871	Immunoglobulin heavy constant mu																			
		P01833	Polymeric immunoglobulin receptor																			
		P0DOX2	Immunoglobulin alpha-2 heavy chain																			
Other immune respons		P55259	Pancreatic secretory granule membrane major GP2																			
		P59665	Neutrophil defensin 1																			
Lipocalin family proteins		P02763	Alpha-1-acid glycoprotein 1																			
		P19652	Alpha-1-acid glycoprotein 2																			
		P80188	Neutrophil gelatinase-associated lipocalin																			
Complement related??		P01024	Complement C3																			
		P0C0L4	Complement C4-A																			
		P02774	Vitamin D-binding protein																			
		P04004	Vitronectin																			
Glycoside Hydrolases		P04746	Pancreatic alpha-amylase																			
		P04745	Alpha-amylase 1A																			
		P19961	Alpha-amylase 2B																			
		P61626	Lysozyme C																			
Proteolityc proteins	Metallloproteases	P15085	Carboxypeptidase A1																			
		P48052	Carboxypeptidase A2																			
		P15086	Carboxypeptidase B																			
	Elastases	P09093	Chymotrypsin-like elastase family member 3A																			
		P08217	Chymotrypsin-like elastase family member 2A																			
		Q99895	Chymotrypsin-C																			
		P08861	Chymotrypsin-like elastase family member 3B																			
		P07477	Trypsin-1																			
		P17538	Chymotrypsinogen B																			
		P00995	Serine protease inhibitor Kazal-type 1																			
	Mucines	P98088	Mucin-5AC																			
		Q9HC84	Mucin-5B																			
		Q6W4X9	Mucin-6																			
		P20142	Gastricsin																			
		P01009	Alpha-1-antitrypsin																			
		P01011	Alpha-1-antichymotrypsin																			
		Q14624	Inter-alpha-trypsin inhibitor heavy chain H4																			
Lipid metabolism-related proteins	Proteolityc proteins	P19835	Bile salt-activated lipase																			
	Lipases	P16233	Pancreatic triacylglycerol lipase																			
		P04118	Colipase																			
		P54317	Pancreatic lipase-related protein 2																			
		P04054	Phospholipase A2																			
		P02749	Beta-2-glycoprotein 1																			
	Lipoproteins	P04114	Apolipoprotein B-100																			
		P02647	Apolipoprotein A-I																			
		P02652	Apolipoprotein A-II																			
		P06727	Apolipoprotein A-IV																			
		P02655	Apolipoprotein C-II																			
		P02656	Apolipoprotein C-III																			
S-100 family proteins		P06702	Protein S100-A9																			
		P05109	Protein S100-A8																			
Coagulation		P02675	Fibrinogen beta chain																			
		P02679	Fibrinogen gamma chain																			
Cell adhesion related-proteins		Q08380	Galectin-3-binding protein																			
		Q6UX06	Olfactomedin-4																			
Regeneration protein family		P05451	Lithostathine-1-alpha																			
		P48304	Lithostathine-1-beta																			
Others		P04217	Alpha-1B-glycoprotein																			
		P00915	Carbonic anhydrase 1																			
		P00918	Carbonic anhydrase 2																			
		P05164	Myeloperoxidase																			
		P32119	Peroxiredoxin-2																			

Table S1: Detailed information of cyst proteomic profiles

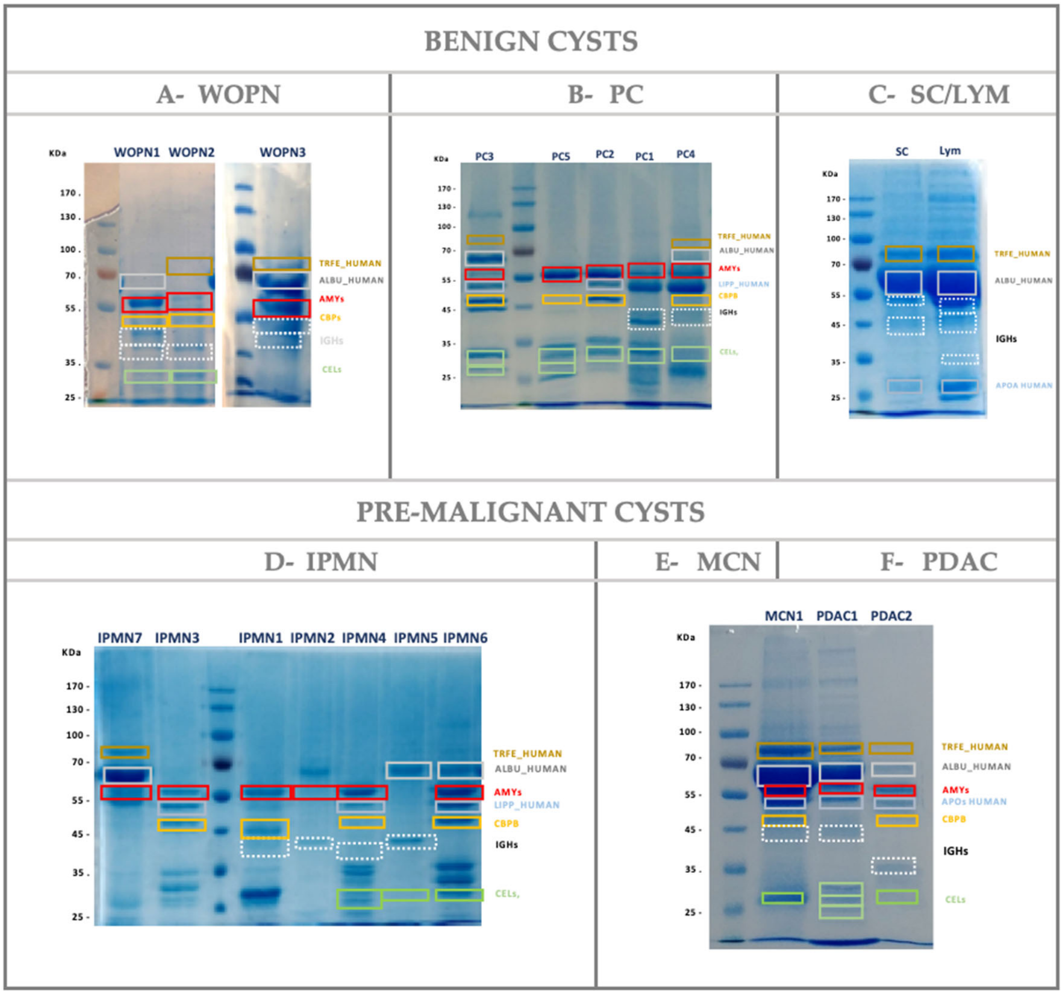

Figure S1: Electrophoresis analysis of proteomic profiles

NON-CYST LESIONS

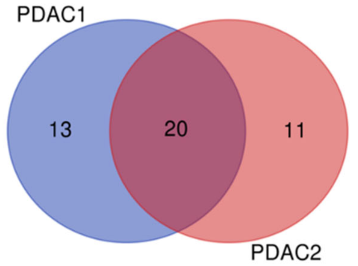

PDAC 1,2	PDAC 1	PDAC 2
**P02763-**Alpha-1-acid glycoprotein 1	**P19652-**Alpha-1-acid glycoprotein 2	**P04745-**Alpha-amylase 1A
**P01023-**Alpha-2-macroglubin	**P01009-**Alpha-1-antitrypsin	**P19961-**Alpha-amylase 2B
**P02768-**Albumin	**P01011-**Alpha-1-antichymotrypsin	**P04746-**Pancreatic alpha-amylase
**P01024**-Complement C3	**P02647-**Apolipoprotein A-I	**P15086-**Carboxypeptidase B
**P69905-**Hemoglobin subunit alpha	**P02652-**Apolipoprotein A-II	**P09093-**Chymotrypsin-like elastase family member 3A
**P68871-**Hemoglobin subunit beta	**P00915-**Carbonic anhydrase 1	**P08861-**Chymotrypsin-like elastase family member 3B
**P02042-**Hemoglobin subunit delta	**P01024-**Complement C3	**P17538-**Chymotrypsinogen B
**P69891-**Hemoglobin subunit gamma-1	**P02100-**Hemoglobin subunit epsilon	**P55259-**Pancreatic secretory granule membrane major glycoprotein GP2
**P02790-**Hemopexin	**P69892-**Hemoglobin subunit gamma-2	**P16233-**Pancreatic triacylglycerol lipase
**P00738-**Haptoglobin	**P01871-** Immunoglobulin heavy constant mu	**P48304-**Lithostathine-1-beta
**P0DOX2-**Immunoglobulin alpha-2 heavy chain	**P0DOY2-**Immunoglobulin lambda constant 2	**P07477-**Trypsin-1
**P0DOX5-**Immunoglobulin gamma-1 heavy chain	**P32119-**Peroxiredoxin-2	
**P01876-**Immunoglobulin heavy constant alpha 1	**P02774-**Vitamin D-binding protein	
**P01859-**Immunoglobulin heavy constant gamma 2		
**P0186-**Immunoglobulin heavy constant gamma 3		
**P01861-**Immunoglobulin heavy constant gamma 4		
**P0DOX7-**Immunoglobulin kappa light chain		
**P01834-**Immunoglobulin kappa constant		
**P0DOX8-**Immunoglobulin lambda-1 light chain		
**P02787-**Serotransferrin		

Figure S2: LC-ESI-MS/MS proteomic content of cyst samples types identification. Common proteins were analyzed via Venn diagrams online tool (http://bioinformatics.psb.ugent.be/beg/tools/venn-diagrams). The different colors represent different groups and numbers inside each set and shared subset indicate the number of identified proteins.

Table S2: Monovariant analysis of TLB parameters of healthy controls and cysts patients (p-value from Wilcoxon test)

Parameter	Healthy Controls (n = 84)	Pancreatic Cysts (n = 14)	p-value
T_{av}	67.96 [67.60;68.39]	68.83 [68.52;68.97]	**<0.001**
G_1	0.26 [0.13;0.35]	0.36 [0.14;0.41]	0.215
WP	7.67 [7.13;8.25]	9.51 [8.78;10.61]	**<0.001**
AUC_{n2}	27.70 [20.08;45.60]	42.16 [29.97;56.02]	**0.047**
AP_{n2}	1.19 [0.63;2.99]	1.93 [0.99;4.88]	0.247
AUC_{n3}	22.80 [20.36;26.06]	24.10 [21.68;30.72]	0.141
AP_{n3}	0.80 [0.58;1.01]	0.79 [0.67;1.09]	0.383
AUC_{n4}	39.64 [28.86;52.37]	32.40 [22.40;44.53]	0.161
AP_{n4}	2.42 [1.43;4.03]	1.40 [0.82;2.36]	0.076
AUC_{n5}	43.06 [39.09;50.85]	44.44 [36.05;86.49]	0.707
AP_{n5}	2.87 [2.25;3.82]	2.85 [1.80;9.51]	0.895
Dv_2	1.19 [1.06;2.05]	0.89 [0.69;3.50]	0.084
Dv_3	1.04 [1.01;1.12]	0.24 [0.15;0.51]	**<0.001**
Dv_4	1.18 [1.04;1.38]	0.63 [0.38;0.89]	**<0.001**
Dv_5	1.04 [1.00;1.17]	0.64 [0.31;2.51]	**0.020**

Note: *median [Q1;Q3]*

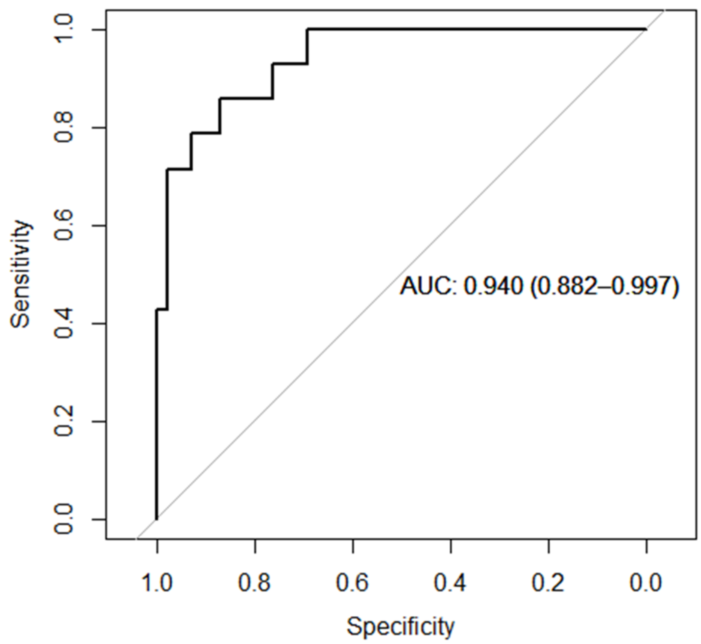

Figure S3: ROC curve illustrating the statistical performance of TLB serum score (healthy vs. cysts). AUC = Area Under Curve (95%CI)

Table S3: Monovariant analysis of TLB parameters of benign and premalignant cysts patients (p-value from t-test or Wilcoxon test depending on the normality character od parameter distribution)

Parameter	Benigno (n = 6)	Premaligno (n = 8)	p-value
T_{av}	68.89 [68.85;69.22]	68.56 [68.41;68.85]	0.071
G_1	0.18 (0.29)	0.32 (0.23)	0.349
WP	10.40 [9.50;11.47]	8.87 [8.58;9.84]	0.0939
AUC_{n2}	37.77 (12.53)	48.53 (18.43)	0.2189
AP_{n2}	1.06 [0.79;2.07]	3.33 [1.78;5.77]	0.1219
AUC_{n3}	26.08 [23.83;30.72]	21.92 [20.60;27.85]	0.156
AP_{n3}	0.96 [0.82;1.09]	0.71 [0.64;0.94]	0.156
AUC_{n4}	41.98 [27.79;47.87]	30.69 [21.51;33.57]	0.366
AP_{n4}	2.19 [1.20;3.08]	1.16 [0.74;1.64]	0.366
AUC_{n5}	38.69 [34.40;58.27]	49.65 [40.10;97.86]	0.439
AP_{n5}	2.29 [1.45;4.82]	3.21 [2.44;13.21]	0.366
Dv_2	0.63 [0.22;1.22]	2.08 [0.75;4.35]	0.156
Dv_3	0.27 [0.14;0.50]	0.23 [0.16;0.51]	1.000
Dv_4	0.55 [0.29;0.98]	0.70 [0.50;0.86]	0.606
Dv_5	0.69 [0.48;0.97]	0.53 [0.26;3.82]	0.897

Note: average (standard deviation) and median [Q1;Q3] are provided depending on the normality character of the parameter distribution.
